# Chrna5 and lynx prototoxins identify acetylcholine super-responder subplate neurons

**DOI:** 10.1016/j.isci.2023.105992

**Published:** 2023-01-14

**Authors:** Sridevi Venkatesan, Tianhui Chen, Yupeng Liu, Eric E. Turner, Shreejoy J. Tripathy, Evelyn K. Lambe

**Affiliations:** 1 Department of Physiology, Temerty Faculty of Medicine, University of Toronto, 1 King’s College Circle, Toronto, ON, Canada; 2 Center for Integrative Brain Research, Seattle Children’s Research Institute, Seattle, WA, USA; 3 Krembil Centre for Neuroinformatics, Centre for Addiction and Mental Health, Toronto, ON, Canada; 4 Department of Psychiatry, University of Toronto, Toronto, ON, Canada; 5 Institute of Medical Science, University of Toronto, Toronto, ON, Canada; 6 Department of Obstetrics and Gynecology, University of Toronto, Toronto, ON, Canada

**Keywords:** Biological sciences, Neuroscience, Molecular neuroscience, Transcriptomics

## Abstract

Attention depends on cholinergic excitation of prefrontal neurons but is sensitive to perturbation of α5-containing nicotinic receptors encoded by *Chrna5*. However, *Chrna5*-expressing (Chrna5+) neurons remain enigmatic, despite their potential as a target to improve attention. Here, we generate complex transgenic mice to probe Chrna5+ neurons and their sensitivity to endogenous acetylcholine. Through opto-physiological experiments, we discover that Chrna5+ neurons contain a distinct population of acetylcholine super-responders. Leveraging single-cell transcriptomics, we discover molecular markers conferring subplate identity on this subset. We determine that Chrna5+ super-responders express a unique complement of GPI-anchored lynx prototoxin genes (*Lypd1*, *Ly6g6e,* and *Lypd6b*), predicting distinct nicotinic receptor regulation. To manipulate lynx regulation of endogenous nicotinic responses, we developed a pharmacological strategy guided by transcriptomic predictions. Overall, we reveal *Chrna5-*Cre mice as a transgenic tool to target the diversity of subplate neurons in adulthood, yielding new molecular strategies to manipulate their cholinergic activation relevant to attention disorders.

## Highlights

• Greatly enhanced cholinergic responses in a subset of *Chrna5*-expressing neurons
• Chrna5+ acetylcholine super-responders comprise molecularly distinct subplate neurons
• Chrna5+ subplate neurons specifically express lynx prototoxins (*Ly6g6e*, *Lypd1*, *Lypd6b*)
• Nicotinic receptor modulation by lynx proteins shapes Chrna5+ cholinergic properties

## Graphical abstract

**Figure:**
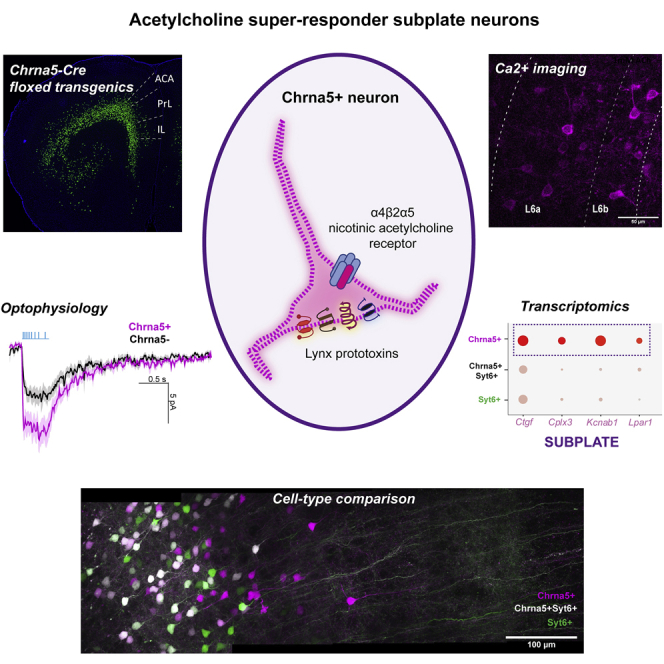

## Introduction

Cholinergic modulation of the medial prefrontal cortex (mPFC) is essential for attention and detection of sensory cues.^1^^,^^2^^,^^3^^,^^4^ Deep-layer pyramidal neurons in the prefrontal cortex (PFC) are critically involved in such executive function^5^^,^^6^^,^^7^ and are robustly excited by acetylcholine,^8^^,^^9^ via nicotinic and muscarinic receptor activation.^10^ The α5 nicotinic receptor subunit encoded by *Chrna5* is specifically expressed in deep-layer pyramidal neurons,^11^^,^^12^ forming high-affinity nicotinic receptors in combination with α4 and β2 subunits. Electrophysiological, behavioral, and genetic evidence in both rodents and humans points to an important role of *Chrna5* expression for nicotinic receptor function, attention, and executive function.

Constitutive deletion of *Chrna5* in mice or knockdown in the adult rat PFC disrupts attention and reduces nicotinic receptor activation by exogenous acetylcholine stimulation in layer 6 neurons.^13^^,^^14^^,^^15^ Optogenetic experiments in *Chrna5*^*−/−*^ mice show that *Chrna5* is required for rapid onset of postsynaptic cholinergic activation and prevents desensitization of the endogenous cholinergic response during prolonged stimulation.^16^ In humans, the non-synonymous rs16969968 (D398N) polymorphism in *Chrna5* is associated with nicotine dependence, schizophrenia, and cognitive impairment.^17^^,^^18^^,^^19^ Nicotinic α4β2α5 receptors with the D398N polymorphism have partial loss of function, attributed to changes in receptor desensitization, calcium permeability, or membrane trafficking.^20^^,^^21^^,^^22^

Despite α5 nicotinic receptor involvement in attention and prefrontal cholinergic activation, systematic characterization of *Chrna5-*expressing (Chrna5+) neurons using modern genetic tools is lacking. Deep-layer neurons include diverse corticothalamic (L6CT), corticocortical, and layer 6b (L6b) populations.^23^^,^^24^^,^^25^ *Chrna5* is predicted to be expressed in L6CT neurons,^8^^,^^26^ which are usually identified by their expression of *Syt6*, a conserved L6CT neuronal marker. *Syt6*-Cre mice have been widely used to characterize prefrontal L6CT neurons and their cholinergic properties.^27^^,^^28^^,^^29^ However, it is unclear whether these are the same neurons expressing *Chrna5.* Characterization of Chrna5+ neurons has been limited by the lack of verified antibodies for the α5 subunit that could be used for post hoc immunostaining. Previous bacterial artificial chromosome (BAC)-transgenic mice labeling Chrna5+ neurons had altered expression of other genes in the tightly linked *Chrna5/a3/b4* gene cluster, limiting their use for functional examination.^30^ This issue was circumvented by disrupting the open reading frames of *Chrna3/b4* in the BAC transgene to generate a *Chrna5*-Cre mouse without misexpression artifacts.^31^

Here, we create compound transgenic mice to investigate how prefrontal Chrna5+ neurons respond to optogenetic release of endogenous acetylcholine. The fast and strong response prompted a multi-approach examination of Chrna5+ neurons together with a control population of *Syt6*-expressing (Syt6+) neurons, a more traditional molecular marker of deep-layer prefrontal cortex. We demonstrate a large fraction of Chrna5+ neurons have distinctive high-affinity cholinergic responses. Single-cell RNA sequencing (RNA-seq) analysis reveals the expression of several subplate markers (*Cplx3*, *Ctgf*, and *Lpar1*) in this Chrna5+ subset, identifying them as subplate neurons born early in development that are critical for establishing thalamocortical connectivity.^32^^,^^33^^,^^34^ Chrna5+ subplate neurons show distinct expression pattern of glycosylphosphatidylinisotol (GPI)-anchored lynx prototoxins (*Ly6g6e*, *Lypd1*, and *Lypd6b*) capable of exerting complex modulation of nicotinic receptor properties.^35^^,^^36^ As predicted from the transcriptomic analysis, our pharmacological manipulations targeting lynx prototoxins successfully alter endogeneous nicotinic responses, revealing cell type-specific lynx regulation of nicotinic receptors.

Recent studies have examined cell type-specific modulation of nicotinic receptors by different lynx prototoxins and the consequences for cortical development and cognition.^37^^,^^38^^,^^39^^,^^40^ Here, we discovered endogenous lynx modulation of nicotinic properties relevant for attention in subplate neurons expressing *Chrna5*. Our work highlights *Chrna5*-Cre mice as a transgenic tool to target acetylcholine super-responder subplate neurons and identifies strategies to fine-tune their cholinergic activation by manipulating GPI-anchored lynx prototoxins.

## Results

### *Chrna5* expression identifies acetylcholine “super-responders”

**Figure 1:**
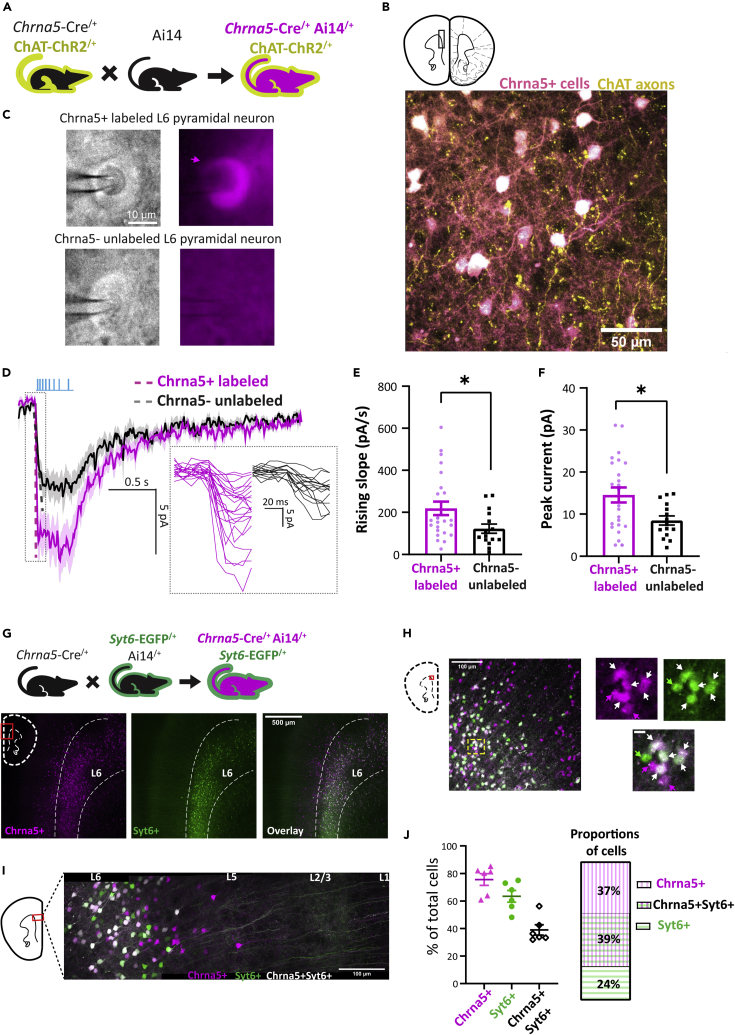
*Chrna5* expression identifies a distinct population of prefrontal neurons with stronger and faster-onset optogenetic cholinergic responses (A) Breeding scheme to obtain triple transgenic *Chrna5*-Cre^/+^Ai14^/+^ChAT-ChR2^/+^ mice expressing tdTomato in *Chrna5*-expressing (Chrna5+) neurons and Channelrhodopsin 2 in cholinergic axons. (B) (*Top*) Schematic of coronal mPFC slice with region of interest, adapted from Paxinos and Franklin.^41^ (*Bottom*) two-photon imaging (3D projection) of tdTomato-labeled Chrna5+ neurons and EYFP-labeled cholinergic axons in layer 6 of mPFC slices. (C) Infrared differential interference contrast (IRDIC) (*left*) and widefield fluorescence (TRITC, *right*) microscopy images of tdTomato-labeled Chrna5+ and unlabeled layer 6 neurons during whole-cell patch clamp electrophysiology. Clearing induced by the pipette is visible. (D) Average light-evoked endogenous cholinergic response of labeled Chrna5+ vs neighboring unlabeled Chrna5- neurons. Dotted lines are the slope of the response onset. (*Inset*) Individual responses are zoomed in to show the onset. (E and F) Bar graph comparing (E) Rising slope and (F) Peak current of endogenous cholinergic responses between labeled Chrna5+ neurons (n = 24 cells) and unlabeled Chrna5- neurons (n = 15 cells, 4 mice). ∗p < 0.05, Unpaired t test. (G) (*Top*) Breeding scheme to obtain triple transgenic *Chrna5*-Cre^/+^Ai14^/+^*Syt6*-EGFP^/+^ mice expressing tdTomato in Chrna5+ neurons and EGFP in Syt6+ neurons. (*Bottom*) Confocal imaging in mPFC slices shows Chrna5+ and Syt6+ neurons distributed in layer 6. (H and I) Confocal (H) and two-photon imaging (I) reveal three populations of neurons: exclusively Chrna5+ neurons which do not express *Syt6*, overlapping Chrna5+Syt6+ neurons which express both markers, and exclusively Syt6+ neurons which do not express *Chrna5.* (J) (*left*) Graph quantifies the percentage of each cell type with respect to all labeled cells per sample. (*Right*) Average proportions of Chrna5+, Chrna5+Syt6+, and Syt6+ neurons. (See Figures S1–S3 for additional information on distribution of Chrna5+ cells across the cortex and additional recordings showing stronger and faster opto-cholinergic responses in Chrna5+ neurons in primary somatosensory cortex.).

To characterize prefrontal Chrna5+ neurons, we generated triple transgenic *Chrna5*-Cre^/+^Ai14^/+^ChAT-ChR2^/+^ mice (Figure 1A) and examined optogenetic cholinergic responses in labeled Chrna5+ neurons. These mice expressed tdTomato in Chrna5+ neurons and channelrhodopsin-2 in cholinergic axons as seen by two-photon imaging (Figure 1B). The location of reporter-labeled Chrna5+ neurons across the brain in *Chrna5*-Cre mice is specific to layer 6 and found across the entire cortical mantle (Figure S1). We recorded endogenous cholinergic responses in labeled Chrna5+ neurons with optophysiology in mPFC slices, using unlabeled pyramidal neurons as a control (Figure 1C). Optogenetic Chrna5+ neurons clearly possessed larger-amplitude cholinergic responses with significantly faster onset compared to control neurons (Figures 1D–1F). The rising slope was significantly larger in Chrna5+ neurons (220 ± 32 pA/s, 24 cells from 4 mice) compared to control (123 ± 22 pA/s, 15 cells; t_(37)_ = 2.19, p = 0.02, unpaired t-test; Cohen’s d: 0.72). Similarly, peak current evoked by optogenetic acetylcholine release was greater in Chrna5+ neurons (15 ± 2 pA) compared to control (8 ± 1 pA, t_(37)_ = 2.52, p = 0.02; Cohen’s d: 0.83), as well as the area of the cholinergic response (t_(37)_ = 2.06, p = 0.046). Intrinsic electrophysiological properties, however, did not differ between Chrna5+ and unlabeled neurons (Table S1). In addition, we determined that Chrna5+ neurons are under presynaptic muscarinic autoinhibitory control, which can be relieved by atropine, resulting in even larger responses (post-atropine response: 194 ± 42% of baseline, n = 6 cells). Our application of the nicotinic antagonist dihydro-β-erythroidine (DHBE) eliminated the optogenetic cholinergic response (post-DHBE response: 3 ± 2% of baseline, n = 4 cells), indicating the relevant nicotinic receptors contain β2 subunits. This characterization of Chrna5+ neurons revealed them to be acetylcholine “super-responders” with stronger and faster onset cholinergic responses distinct from other deep-layer pyramidal neurons. We next examined whether Chrna5+ neurons in other cortical regions are also acetylcholine super-responders. Chrna5+-labeled neurons deep in layer 6 of primary somatosensory cortex (SSp) were also found to show significantly stronger and faster onset nicotinic responses to optogenetic acetylcholine release (Figure S2) confirming that *Chrna5*-labeling identifies acetylcholine super-responders in multiple cortical areas.

To further probe the distinct cholinergic responses of prefrontal Chrna5+ neurons, we examined factors differentiating these cells from neurons labeled by *Syt6* expression (Syt6+), which has hereto been the predominant marker of layer 6 corticothalamic neurons used to characterize their function during PFC-dependent tasks.^27^^,^^28^^,^^42^^,^^43^ Because the extent of overlap between Chrna5+ and Syt6+ deep-layer pyramidal neuron populations was unclear, we adopted an imaging strategy to visualize the distribution of Chrna5+ and Syt6+ neurons in the PFC and determine the exact proportion of distinctive nonoverlapping Chrna5+ neurons. We generated a compound transgenic *Chrna5*-Cre^/+^Ai14^/+^*Syt6*-EGFP^/+^ mouse to simultaneously express tdTomato in Chrna5+ neurons and EGFP in Syt6+ neurons and performed confocal and two-photon imaging of the endogenous fluorescence of these reporters in mPFC brain slices. Chrna5+ and Syt6+ neurons were both present primarily in layer 6, with a few Chrna5+ neurons in layer 5 (Figures 1G and 1I). Figure 1J (left) shows the proportion of all cells expressing tdTomato (*Chrna5*), GFP (*Syt6*)*,* or both, as a percentage of the total number of fluorescent cells in that region. Closer investigation confirmed the existence of a substantial proportion of exclusively Chrna5+ neurons (37% of all labeled cells) which do not express *Syt6*, in addition to overlapping Chrna5+Syt6+ neurons (39%) which express both markers, and exclusively Syt6+ neurons (24%) which do not express *Chrna5* (N = 4 mice, Figures 1H and 1J). Thus, nearly half of all Chrna5+ neurons are not labeled by *Syt6* expression and would have been excluded in previous studies using *Syt6*-Cre mice. Furthermore, Chrna5+ neurons are found even in primary visual cortex, where *Syt6* labeling is greatly reduced^28^^,^^44^ (Figure S3).

### Distinct Chrna5+ neurons with highly resilient nicotinic receptor responses

**Figure 2:**
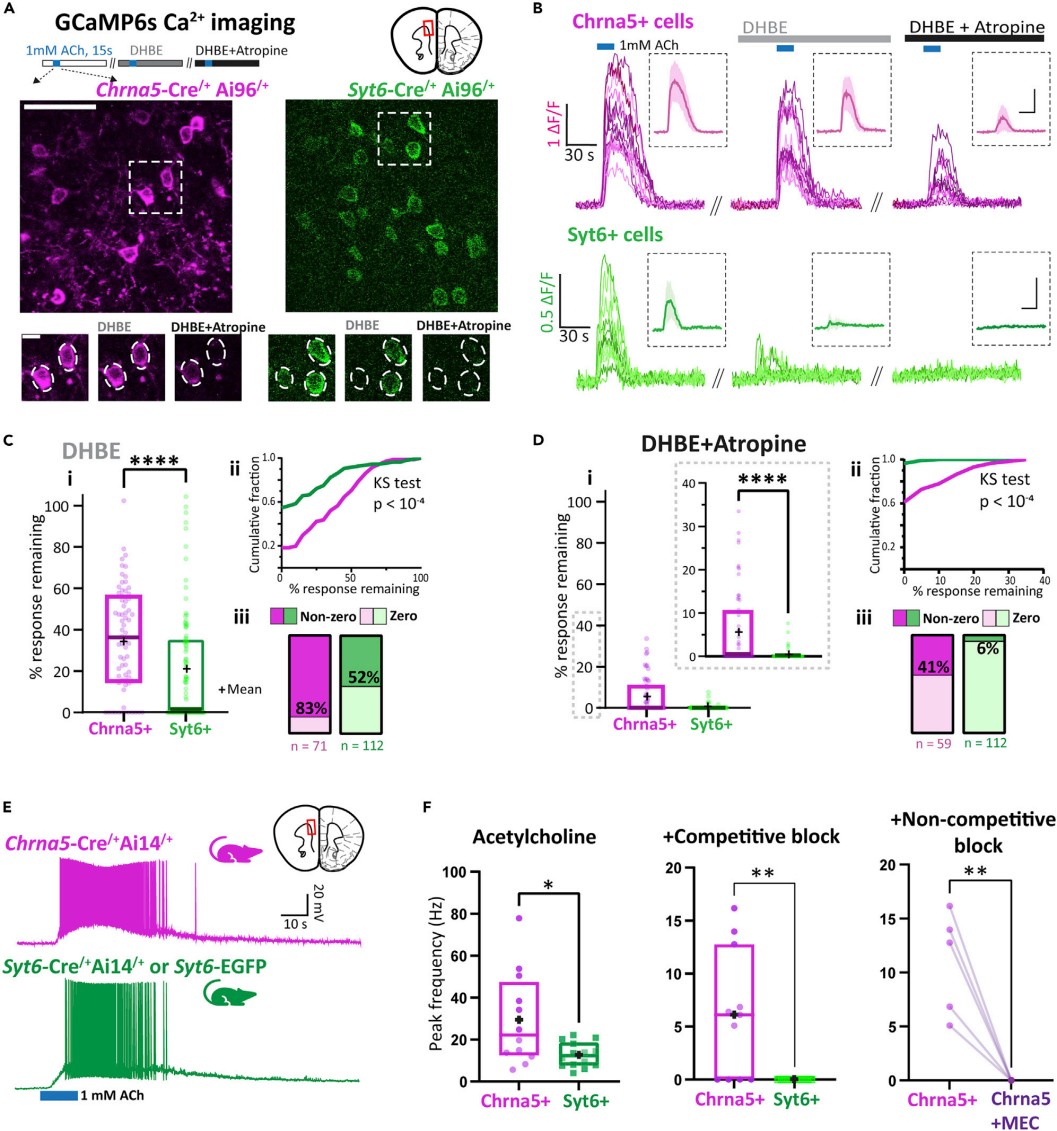
Calcium imaging in Chrna5+ and Syt6+ populations reveals a distinct subset of Chrna5+ neurons with resilient nicotinic responses (A) (*Top*) Two-photon Ca^2+^ imaging in prefrontal brain slices from *Chrna5*-Cre^/+^Ai96^/+^ (*left*) and *Syt6*-Cre^/+^Ai96^/+^ mice (*right*) showing acetylcholine-evoked GCaMP6s responses in Chrna5+ and Syt6+ neurons respectively (scale 50 μm). (*Bottom*) Acetylcholine-evoked GCaMP6s signals were sequentially recorded after application of competitive nicotinic antagonist DHBE and addition of muscarinic antagonist atropine (scale 10 μm). Examples of Chrna5+ or Syt6+ neuronal responses to acetylcholine are illustrated in Video S1 (Chrna5+) and Video S2 (Syt6+). (B) Normalized fluorescence signal (ΔF by F) evoked by acetylcholine in individual Chrna5+ and Syt6+ neurons in a brain slice at baseline (*left*), after DHBE (*middle*), and after DHBE + Atropine (*right*). (Inset, average response and SD. Scale: same as main figure). (C and D) i, Boxplot shows the percentage of response remaining after the application of (C) DHBE and (D) DHBE + Atropine (Inset shows the same boxplot with a restricted y axis, ‘+’ symbols denote respective means). Responses were quantified by the area under the ΔF/F curve (n = 71 neurons, 6 mice for Chrna5+, and 112 neurons, 7 mice for Syt6+, ∗∗∗∗p < 10^−4^, Mann-Whitney test). (C and D) ii, Cumulative frequency distribution of the percentage response remaining after (C) DHBE and (D) DHBE + Atropine (p < 10^−4^, Kolmogorov-Smirnov test). C-D iii, Proportion of cells showing zero and nonzero responses after (C) DHBE and (D) DHBE + Atropine (p < 10^−4^ for both C & D, Fisher’s exact test). (E) Current clamp responses evoked by 1 mM acetylcholine (15s) in fluorescently labeled Chrna5+ and Syt6+ layer 6 neurons patched in mPFC slices from mice *Chrna5*-Cre^/+^Ai14^/+^ and *Syt6*-Cre^/+^Ai14^/+^ or *Syt6*-EGFP mice, respectively. (F) Peak spike frequency in Chrna5+ and Syt6+ neurons evoked by acetylcholine (*left*) and in the presence of competitive nicotinic antagonist DHBE and atropine (*middle;* ∗∗p < 0.01,∗p < 0.05, unpaired t test). Residual response remaining after DHBE + Atropine in Chrna5+ neurons is blocked by noncompetitive nicotinic antagonist mecamylamine (*right,* ∗∗p < 0.01, paired t test). (See Figure S4 for localization of Chrna5+ neurons in PFC with resilient nicotinic responses in layer 6b.).

To elucidate the distinct subset of Chrna5+ neurons not found with Syt6-labeling approaches, we measured acetylcholine-evoked signals in multiple neurons simultaneously with *ex vivo* GCaMP6s Ca^2+^ imaging. We generated transgenic mice expressing GCaMP6s in either Chrna5+ (*Chrna5*-Cre^/+^/Ai96^/+^) or Syt6+ (*Syt6*-Cre^/+^/Ai96^/+^) neurons and performed two-photon imaging of mPFC layer 6 (Figure 2A). We measured Ca^2+^ signals evoked by acetylcholine (1 mM, 15 s) in Chrna5+ (Video S1) and Syt6+ neurons (Video S2). To pharmacologically interrogate these populations, we measured changes in the Ca^2+^ signal and proportions of acetylcholine-responsive neurons (Figures 2A and 2B). Application of the competitive nicotinic antagonist DHBE (10 μM, 10 min) left residual acetylcholine-evoked Ca^2+^ signals in Chrna5+ neurons (35 ± 3% of baseline, n = 71 cells, 6 mice; Figure 2C i) that were greater than those in Syt6+ neurons (21 ± 3% of baseline, n = 112 cells, 7 mice; Mann-Whitney U = 2400, p < 10^−4^). The cumulative distribution of responses remaining after DHBE was significantly right-shifted in Chrna5+ neurons compared to Syt6+ neurons (Figure 2C **ii**, Kolmogorov-Smirnov D = 0.37, p < 10^−4^). A majority of Chrna5+ neurons (∼83%) still showed acetylcholine-evoked responses after DHBE, whereas fewer Syt6+ neurons (∼50%) retained their responses, with a complete elimination of acetylcholine-evoked responses in the rest (Figure 2C **iii**, Fisher’s exact test: p < 10^−4^). Yet, the addition of muscarinic antagonist atropine did not attenuate the striking differences between Chrna5+ and Syt6+ neurons, raising the possibility of an underlying nicotinic mechanism (Figure 2D). In the presence of DHBE + atropine, a subset of Chrna5+ neurons still showed substantial acetylcholine-evoked Ca^2+^ signals (6 ± 1% of baseline; Figure 2D i), whereas almost all Syt6+ neurons’ responses were completely blocked (0.2 ± 0.1% of baseline; Mann-Whitney U = 2083, p < 10^−4^) as seen from the cumulative distribution (Figure 2D **ii**, Kolmogorov-Smirnov D = 0.36, p < 10^−4^). The proportion of Chrna5+ and Syt6+ neurons showing acetylcholine-evoked responses after DHBE + Atropine was significantly different (41% Chrna5+ vs 6% Syt6+, Fisher’s exact test: p < 10^−4^, Figure 2D **iii**). Chrna5+ neurons resilient to DHBE + Atropine within a slice appear to be localized to layer 6b (Figure S4). Because DHBE is a competitive antagonist, it can be outcompeted by exogenous acetylcholine at high-affinity nicotinic receptors. Therefore, we hypothesized that the resilient exogenous acetylcholine-evoked responses would require noncompetitive antagonist.

Video S1. Video shows Chrna5+ neurons in mPFC slices from Chrna5-Cre/^+^Ai96/^+^ mice responding to exogenous application of 1 mM acetylcholine with an increase in GCaMP6s fluorescence signal, related to Figure 2

Video S2. Video shows Syt6+ neurons from Syt6-Cre/^+^Ai96/^+^ mice responding to exogenous application of 1 mM acetylcholine with an increase in GCaMP6s fluorescence signal, related to Figure 2

To address the hypothesis of higher-affinity nicotinic binding and its consequences for spiking in these -neurons, we switched to whole-cell recording in individual Chrna5+ and Syt6+ neurons. We recorded current clamp responses to acetylcholine (1 mM, 15 s) in labeled Chrna5+ and Syt6+ neurons from *Chrna5*-Cre^/+^Ai14^/+^ and *Syt6*-Cre^/+^Ai14^/+^ or *Syt6*-EGFP mice, respectively (Figure 2E). Chrna5+ neurons showed stronger acetylcholine-evoked firing, attaining significantly higher peak firing frequency (29 ± 6 Hz, n = 12 cells, 6 mice; t_(24)_ = 2.74, p = 0.01, unpaired t test; Cohen’s d: 1.08) compared to Syt6+ neurons (13 ± 2 Hz, n = 14 cells, 5 mice; Figure 2E). The intrinsic electrophysiological properties of Chrna5+ and Syt6+ neurons did not show statistically significant differences (Table S2). We next examined the sensitivity of acetylcholine-evoked firing to competitive nicotinic receptor block by DHBE in the presence of atropine. Acetylcholine-evoked firing was completely eliminated in all Syt6+ neurons (Figure 2F) whereas a large subset of Chrna5+ neurons (7/11) retained their ability to respond to acetylcholine (average peak firing rate in Chrna5+ neurons: 6 ± 2 Hz; t_(19)_ = 3.22, p = 0.004, unpaired t test) demonstrating similar resilience to competitive nicotinic receptor block as observed with Ca^2+^ imaging. We used the noncompetitive nicotinic receptor blocker mecamylamine^45^^,^^46^ to test our hypothesis that nicotinic receptors in this Chrna5+ subset were higher affinity and therefore allowed exogenous acetylcholine to outcompete DHBE. The addition of 5 μM mecamylamine was sufficient to eliminate acetylcholine-evoked firing in all the Chrna5+ neurons that were resilient to competitive nicotinic block (t_(4)_ = 5.14, p = 0.007, paired t test; Figure 2F). Together, our Ca^2+^ imaging and electrophysiology experiments revealed the existence of a distinct subset of Chrna5+ neurons dissimilar to Syt6+ neurons, with high-affinity nicotinic responses resilient to competitive nicotinic antagonism.

### Single-cell transcriptomics identifies Chrna5+ subplate neurons with lynx genes

**Figure 3:**
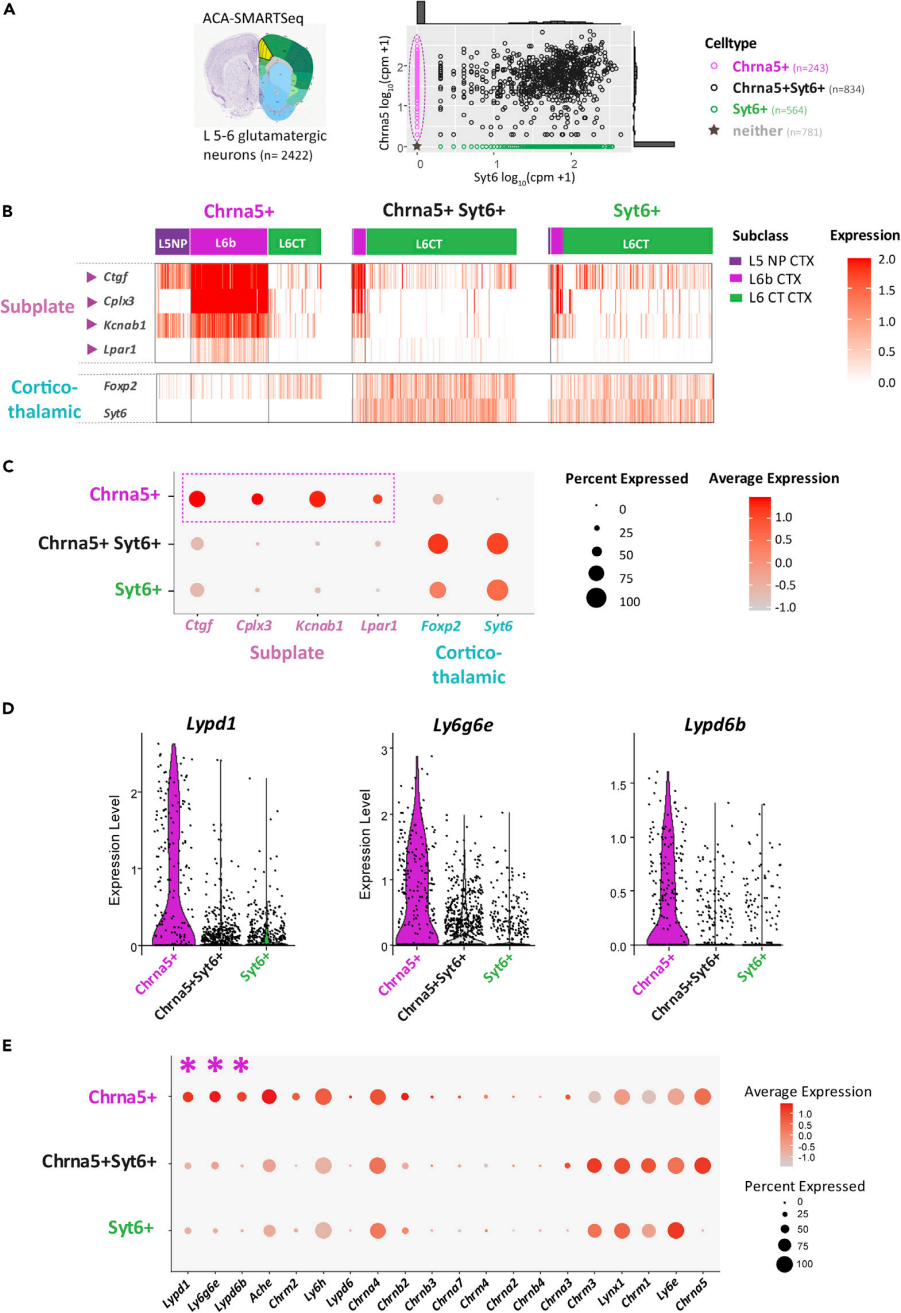
Single-cell transcriptomic analysis reveals Chrna5+ subset to span subplate neuron populations with differential expression of lynx prototoxin genes (A) Single-cell RNA-seq data for 2422 L5-6 glutamatergic neurons in the anterior cingulate cortex (ACA, shown in schematic on the left) was obtained from publicly available datasets (Allen Institute, SMARTSeq ACA and MOP (2018)). (*Right*) Scatterplot showing *Chrna5* vs *Syt6* expression in log_10_ (Copies per million +1) for each neuron, with the frequency distribution shown on the corresponding axes. Neurons were classified into Chrna5+, Chrna5+Syt6+, and Syt6+ groups based on their expression of *Chrna5* and *Syt6* genes. Cells which expressed neither gene were excluded from subsequent analyses. (B) The major neuronal subclasses within Chrna5+, Chrna5+Syt6+, and Syt6+ groups is indicated by the colorbar on top. NP- Near projecting, CT- Corticothalamic. Heatmap shows expression of subplate and corticothalamic marker genes in each cell in all 3 groups. (C) Dotplot shows summary of subplate and corticothalamic marker expression in Chrna5+, Chrna5+Syt6+, and Syt6+ groups. Dot size indicates the percentage of cells within each group expressing that gene, and color of the dot indicates average expression level relative to other groups. Chrna5+ neurons highly express multiple subplate-marker genes, but not corticothalamic markers. (D) Violin plots show expression of Lynx prototoxin genes *Ly6g6e*, *Lypd1* (Lynx2), and *Lypd6b* which show highest fold change between Chrna5+ and Chrna5+Syt6+ neurons. (E) Dotplot shows expression of major genes known to modulate cholinergic function, including nicotinic, muscarinic subunits, acetylcholinesterase, and lynx prototoxins in Chrna5+, Chrna5+Syt6+, and Syt6+ neurons. Genes are ordered by decreasing fold change in expression. Dot size indicates the percentage of cells within each group expressing that gene, and color of the dot indicates average expression level relative to other groups. Fold change of all the genes shown in this dotplot are listed in Table S3. (See Figure S5 for transcriptomic analysis of Chrna5+ neurons in other cortical areas showing conserved subplate markers and lynx prototoxin gene expression across cortical regions).

To determine the molecular identity of distinct Chrna5+ neurons with enhanced cholinergic responses, we pursued single-cell RNA-seq. We extracted gene expression data of L5-6 glutamatergic neurons (n = 2422 cells) in the mouse anterior cingulate cortex from the Allen Institute single-cell RNA-seq databases.^47^^,^^48^^,^^49^ We classified these deep-layer pyramidal neurons into 3 groups: those expressing only *Chrna5* (Chrna5+, n = 243), both *Chrna5* and *Syt6* (Chrna5+Syt6+, n = 834), or only *Syt6* (Syt6+, n = 564) (Figure 3A). 781 cells showed no expression of *Chrna5* or *Syt6* and consisted primarily of L6 intratelencephalic cells which have been previously shown to have purely muscarinic M2/M4-mediated hyperpolarizing cholinergic responses.^16^^,^^50^ We focused on the Chrna5+, Syt6+, and Chrna5+Syt6+ groups to examine their transcriptomic differences. Prior work has shown that deep cortical neurons can be separated into L6b, L5, and L6CT subclasses by the expression of characteristic markers and hierarchical clustering.^49^ Single-cell analysis revealed that the Chrna5+ group primarily included L6b (44%), L5 near-projecting (L5NP, 19%), and L6CT neurons (30%), whereas the Chrna5+Syt6+ and Syt6+ groups were predominantly composed of L6CT neurons (>90%) (Figure 3B). We further examined the expression of marker genes in these respective groups to validate our cell classification. Chrna5+ neurons showed distinctive expression of several marker genes, *Ctgf, Cplx3, Kcnab1,* and *Lpar1* (Figures 3B and 3C) associated with subplate neurons.^32^^,^^51^ Subplate neurons are early born and vital for brain development, leaving L6b neurons as descendants in adulthood.^33^^,^^34^^,^^52^ Notably, the highest fold enrichment among all differentially expressed genes in Chrna5+ neurons was found for subplate markers *Ctgf* (fold change, 5.69) and *Cplx3* (3.81) (Table 1). All differentially expressed genes are listed in Table S4. Overall, Chrna5+ neurons including both L5NP and L6b subpopulations highly express subplate-marker genes. In contrast, *Syt6-*expressing Chrna5+Syt6+ and Syt6+ groups are only enriched in the corticothalamic markers *Foxp2* and *Syt6*, consistent with their corticothalamic subtype. These results support our imaging, electrophysiological, and pharmacological results suggesting the exclusive Chrna5+ population is a distinct cell type from typical L6CT Syt6+ neurons.

**Table 1:** Top 20 differentially expressed genes between Chrna5+ and Chrna5+Syt6+ neurons

Genes	p value	Chrna5+ cells (Proportion)	Chrna5+Syt6+ cells (Proportion)	Adjusted p value	Fold change (Chrna5+/Chrna5+Syt6)
***∗Cplx3***	1.16E-43	0.535	0.149	3.37E-39	5.69
***∗Ctgf***	1.60E-28	0.77	0.568	4.65E-24	3.81
*Ptn*	3.68E-28	0.864	0.758	1.07E-23	3.60
*∗****Tmem163***	2.12E-44	0.51	0.129	6.16E-40	2.58
*Lypd1*	3.40E-23	0.593	0.381	9.88E-19	2.55
***∗Kcnab1***	3.81E-74	0.741	0.191	1.11E-69	2.51
*Serpini1*	1.54E-50	1	0.993	4.49E-46	2.50
*Hpcal1*	2.48E-27	0.658	0.39	7.20E-23	2.47
*Nrsn2*	1.89E-30	0.864	0.767	5.50E-26	2.28
*Ntm*	1.83E-56	0.889	0.59	5.31E-52	2.21
*Tshz2*	2.82E-16	0.535	0.327	8.20E-12	2.17
*Etv1*	5.70E-16	0.395	0.186	1.66E-11	2.16
*Rcn2*	5.18E-46	0.996	0.984	1.51E-41	2.14
*Olfm3*	1.67E-44	0.897	0.716	4.86E-40	2.11
*Crtac1*	5.50E-46	0.728	0.347	1.60E-41	2.04
***∗Ly6g6e***	2.13E-25	0.671	0.434	6.20E-21	2.03
*Adk*	6.31E-29	0.922	0.922	1.83E-24	2.00
*Trp53i11*	2.73E-60	0.724	0.222	7.94E-56	1.93
*Cd164*	6.72E-18	0.897	0.882	1.95E-13	1.92
*Dner*	2.35E-45	0.712	0.326	6.82E-41	1.85

Top 20 differentially expressed genes determined by the FindMarkers function on Seurat ordered by decreasing fold change. Several known subplate neuron markers (highlighted by ∗) are the highest enriched genes in Chrna5+ neurons. All differentially expressed genes are listed in Table S4.

To identify molecular changes predictive of Chrna5+ nicotinic “super-responders”, we examined differential expression of genes that exert effects on postsynaptic cholinergic responses (Figures 3D and 3E). We selected cholinergic receptor genes (nicotinic *Chrna5*-*2, Chrna7, Chrnb2-4,* and muscarinic subunits *Chrm1-4*), acetylcholinesterase (*Ache*), and members of the family of genes that encode lynx proteins (*Ly6e, Ly6h, Ly6g6e, Lynx1, Lypd1, Lypd6,* and *Lypd6b*) known to allosterically modulate nicotinic receptor responses.^35^ We found substantial and highly significant changes in the expression of three lynx prototoxins *Lypd1*, *Ly6g6e*, and *Lypd6b* (Figure 3D). While both Chrna5+ and Chrna5+Syt6+ populations express *Chrna5,* there was slightly higher expression of *Chrna5* (25% increase) as well as lower expression of the inhibitory muscarinic receptor *Chrm2* (20% decrease) in Chrna5+Syt6+ neurons. There were no significant differences in other nicotinic and muscarinic subunit expression between the two groups. Acetylcholinesterase, the enzyme that breaks down acetylcholine was also highly expressed (50% increase) in Chrna5+ neurons, which may benefit their nicotinic responses by protecting receptors from overactivation and desensitization. Of note, *Chrnb2* expression does seem to be in fewer cells than expected (Figure 3E), which can be attributed to sparse detection of *Chrnb2* levels by scRNA-seq approaches.^53^^,^^54^ The fold change of the genes in Figure 3E between Chrna5+ and Chrna5+Syt6+ neurons is shown in Table S3. Notably, the top three genes with the highest fold change in Chrna5+ neurons were the GPI-anchored lynx prototoxins: Lynx2 encoded by *Lypd1* (fold change: 2.55), *Ly6g6e* (2.03), and *Lypd6b* (1.51). The distinct pattern of expression of specific lynx proteins in Chrna5+ neurons suggests unexpectedly complex endogenous control of nicotinic responses in these prefrontal subplate neurons.

Transcriptomic examination of Chrna5+ neurons in other cortical regions including primary motor (MOp), somatosensory (SSp), and visual cortices (VISp) also confirmed that Chrna5+ neurons consist of L6b and L5/6NP neurons expressing subplate-marker genes, in addition to the lynx modulatory proteins as described above in the PFC (Figure S5). Therefore, *Chrna5* expression identifies a conserved population of deep-layer 6 neurons with subplate identity across multiple cortical regions that display specialized expression of lynx proteins to modulate nicotinic receptors.

### Perturbing native prefrontal cortical lynx-modulation of optogenetic nicotinic responses

**Figure 4:**
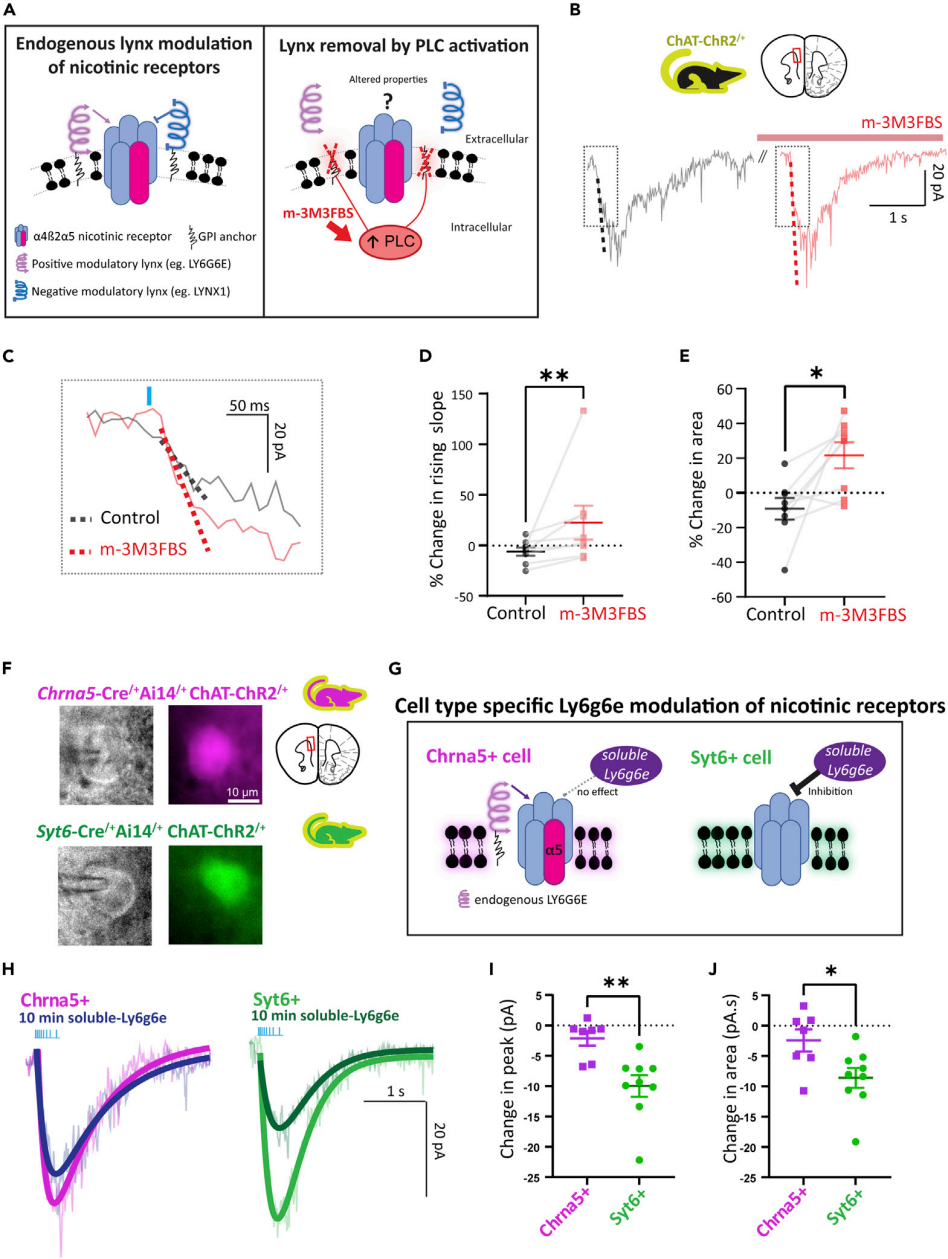
Regulation of optogenetic nicotinic responses by endogenous GPI-anchored lynx proteins and cell type-specific effects of recombinant Ly6g6e (A) Schematic of nicotinic receptor environment showing endogenous GPI-anchored lynx proteins exerting positive and negative modulation of nicotinic receptors. The compound m-3M3FBS activates PLC, cleaving the GPI anchor and perturbing lynx-mediated modulation of nicotinic responses. (B) Optogenetic nicotinic responses in prefrontal deep-layer pyramidal neurons from ChAT-ChR2 mice before and after treatment with m-3M3FBS (5 min). (C) PLC activation significantly increased the rising slope of optogenetic nicotinic responses. Percent change in. (D) Rising slope and (E), Area of nicotinic response in control and after PLC activation. (∗p < 0.05, ∗∗p < 0.01, Wilcoxon matched-pairs test). (F) IRDIC (*left*) and widefield fluorescence (*right*) images of tdTomato-labeled Chrna5+ (*top*) and Syt6+ (*bottom*) neurons during whole-cell patch clamp electrophysiology in *Chrna5*-Cre^/+^Ai14^/+^ChAT-ChR2^/+^ and *Syt6*-Cre^/+^Ai14^/+^ChAT-ChR2^/+^ mouse brain slices, respectively. (G) Schematic summarizing predicted and observed effects of recombinant water-soluble ly6g6e on Chrna5+ and Syt6+ neuronal nicotinic receptors. (H–J), Optogenetic nicotinic responses are reduced in amplitude following 10 min application of soluble ly6g6e in Syt6+ but not Chrna5+ neurons. Change in peak current (I) and area of the nicotinic response (J) of Chrna5+ vs Syt6+ neurons (∗p < 0.05, ∗∗p < 0.01, Unpaired t test).

To examine whether the molecular machinery of deep-layer prefrontal neurons endows them with greater dynamic range in responding to acetylcholine, we sought to experimentally perturb endogenous lynx modulation. Members of the lynx-family are GPI-anchored (Figure 4A), and work on cell expression systems^36^ suggests these anchors can be cleaved via activation of phospholipase C (PLC). These experiments are important because the potential impact of GPI cleavage on nicotinic responses in a native system is not well understood. We hypothesized that perturbing lynx-mediated control could affect endogenous nicotinic properties in a complex manner (Figure 4A) since both positive (e.g., Ly6g6e) and negative (e.g., Lynx1) modulatory lynx proteins are expressed. To cleave GPI-anchored proteins, we used the PLC activator compound m-3M3FBS.^55^^,^^56^^,^^57^^,^^58^ Nicotinic responses of deep-layer pyramidal neurons from ChAT-ChR2 mice to optogenetic acetylcholine release were recorded in the continuous presence of atropine before and after treatment with m-3M3FBS (25 μM, 5 min; Figure 4B). The rising slope of the nicotinic responses showed a significant increase after m-3M3FBS treatment (23 ± 17%; Paired Cohen’SD = 0.83; p = 0.008, Wilcoxon matched-pairs test), compared to the baseline change observed in the same cells prior to PLC activation (−6 ± 4%; 8 cells, 6 mice; Figures 4C and 4D). This increase was not observed with the inactive ortholog o-3M3FBS that does not activate PLC (paired Cohen’s d = 0.09, p = 0.625, Wilcoxon matched-pairs test, data not shown). The area under the nicotinic response known as charge transfer also showed a statistically significant increase following PLC activation (22 ± 7%; Cohen’s d = 1.68; p = 0.016, Wilcoxon matched-pairs test; Figure 4D), compared to baseline change (−9 ± 6%). Thus, PLC activation causes a specific increase in nicotinic receptor responses, presumably due to cleavage of inhibitory GPI-anchored prototoxins such as Lynx1.

To test the transcriptomic prediction that cell type-specific differences in lynx modulation lead to different cholinergic properties, we obtained purified water-soluble recombinant Ly6g6e protein and examined its effects on optogenetic nicotinic responses in labeled Chrna5+ and Syt6+ neurons (Figure 4F). These experiments were conducted in *Chrna5*-Cre^/+^Ai14^/+^ChAT-ChR2^/+^ and *Syt6*-Cre^/+^Ai14^/+^ChAT-ChR2^/+^ mice. We hypothesized that the modulation of Chrna5+ neuronal nicotinic receptors by endogenous Ly6g6e would occlude the effect of exogenous soluble Ly6g6e, whereas Syt6+ neurons would be altered by exposure to the exogenous Ly6g6e (Figure 4G). Consistent with this hypothesis, we found that 10 min application of soluble Ly6g6e did not significantly alter the amplitude of optogenetically evoked nicotinic responses in labeled Chrna5+ neurons (change in peak = −2.1 ± 1.2 pA, t_(6)_ = 1.79, p = 0.12, paired t test). However, in labeled Syt6+ neurons lacking endogenous expression of *Ly6g6e*, exogenous application of soluble Ly6g6e caused a significant decrease in the amplitude (change in peak = −10 ± 1.8 pA, t_(8)_ = 5.60, p < 0.001, paired t test; Figures 4H and 4I). The change in peak and area of the nicotinic responses caused by solube Ly6g6e was significantly different between Chrna5+ and Syt6+ neurons (change in peak: t_(14)_ = 3.43, p = 0.004; change in area: t_(14)_ = 2.53, p = 0.024, unpaired t test; Figures 4I and 4J). Of note, soluble and endogenous GPI-anchored prototoxins are known to have opposite effects on nicotinic receptors, and the exact direction of endogenous modulation of nicotinic receptors by different lynx proteins is still debated.^38^^,^^59^^,^^60^ The key outcome of this experiment is the difference in the Ly6g6e modulation of Chrna5+ and Syt6+ neurons, not the direction. We conclude that Chrna5+ neurons exert specialized molecular control over their nicotinic receptors, shaping their fate as acetylcholine super-responders.

## Discussion

**Figure 5:**
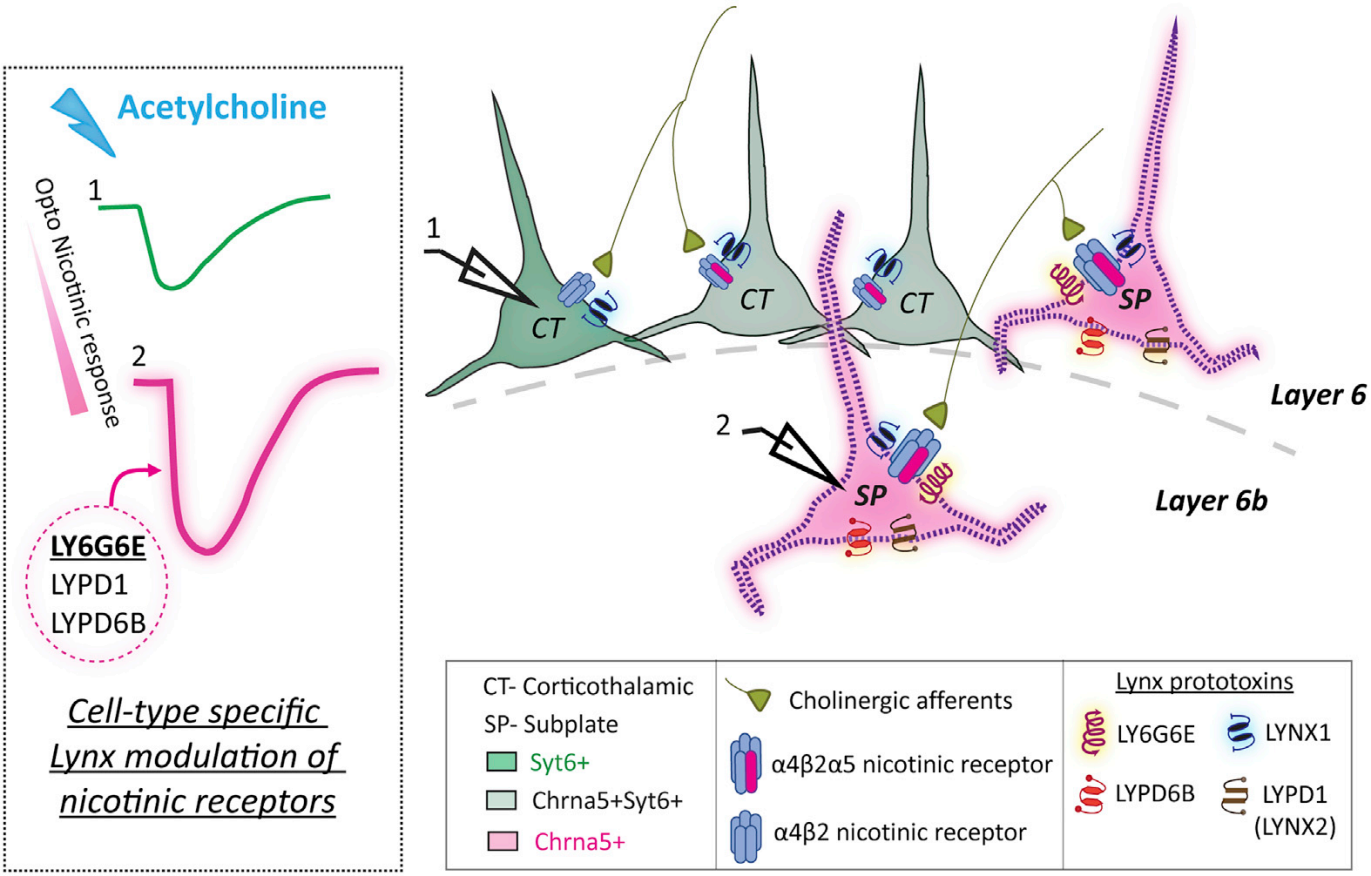
Graphical summary Deep-layer pyramidal neurons can be divided into three groups (Chrna5+, Chrna5+Syt6+, Syt6+) by their expression of *Chrna5* and *Syt6* genes. The subset of *Chrna5*-expressing neurons without *Syt6* expression are molecularly distinct and comprise subplate neurons, whereas *Syt6*-expressing neurons are of the corticothalamic subtype. Nicotinic receptors in these neurons are under complex regulation by endogenous lynx prototoxins. Inhibitory prototoxin gene *Lynx1* is expressed uniformly in all neurons, whereas Chrna5+ subplate neurons additionally have specific expression of *Ly6g6e*, *Lypd1*, and *Lypd6b* prototoxin genes. These Chrna5+ subplate neurons in layer 6b show enhanced α5 subunit nicotinic receptor-mediated cholinergic responses that are differently modulated by specific lynx prototoxins.

Our work examines the effects of GPI-anchored lynx prototoxins on native nicotinic receptor-mediated optogenetic responses, advancing from work on heterologous expression systems. These results are a first step in showing how endogenous lynx regulation of nicotinic responses can act in a complex cell type-specific fashion leading to specialized cholinergic properties in a subset of neurons. Overall, our study reveals a previously uncharacterized population of Chrna5+ subplate neurons in the prefrontal cortex that are exquisitely sensitive to acetylcholine, with differential expression of several lynx prototoxin genes that allow flexible tuning of their high-affinity nicotinic responses (Figure 5).

### Specialized cholinergic properties of Chrna5+ neurons

While important for attention, the neurophysiological impact of the auxiliary α5 nicotinic subunit in its native neuronal environment has remained beyond the reach of previous work. The contributions of α5 to high-affinity nicotinic receptors have been extrapolated based on results of cell system experiments and work on rodents deleted for *Chrna5*.^13^^,^^15^^,^^16^^,^^22^^,^^61^ Here, *Chrna5*-Cre mice allowed us to affirmatively demonstrate that neurons expressing the α5 nicotinic subunit respond faster and more strongly to endogenous acetylcholine. This cholinergic heterogeneity among layer 6 neurons prompted a larger-scale comparison between Chrna5+ neurons and a well-defined layer 6 population labeled by *Syt6*.^42^ These experiments revealed a subset of Chrna5+ acetylcholine “super-responders” with high-affinity nicotinic responses that were not found in Syt6+ neurons.

### Heterogeneity of cell types expressing Chrna5

Previously, the deep-layer cell types expressing *Chrna5* were uncharacterized and generally thought to include L6CT neurons.^8^ Investigation of L6CT neurons have relied on *Syt6*-Cre and *Ntsr1*-Cre mouse lines that label similar sets of neurons,^42^^,^^43^^,^^62^ with only *Syt*6-Cre mice successfully labeling this population in PFC.^28^ L6CT neurons labeled by *Syt6* or *Ntsr1* expression are excited by acetylcholine,^29^^,^^63^ but the degree to which their nicotinic response relied on *Chrna5* expression was unclear. Strikingly, we reveal that acetylcholine super-responders with high-affinity nicotinic receptors are from the population of Chrna5+ neurons without *Syt6-*expression. Transcriptomic analysis demonstrates that majority of these likely Chrna5+ “super-responders” arise from L5 near-projecting and L6b neurons, populations that express multiple markers linking them to the developmental subplate. These enigmatic cells are remnants of earliest-born cortical neurons that serve as a relay for establishing connections between cortex and thalamus.^64^^,^^65^ Subplate neurons receive cholinergic inputs at birth,^66^ highlighting their role in developmental cholinergic modulation.

### Advantages of Chrna5 as a marker for subplate cells

In contrast to L6CT neurons, subplate neurons remain relatively uncharacterized due to the lack of transgenic mice to definitively label the diverse subtypes and inaccessibility of the available lines for *in vivo* targeting. Transcriptomic analysis (Figure 5, Table 1) suggests that the Chrna5+ population is enriched for known subplate markers *Ctgf* (connective tissue growth factor), *Cplx3* (complexin 3), *Kcnab1,* and *Lpar1*.^32^^,^^67^^,^^68^ Significantly, the lynx prototoxin and nicotinic receptor modulator *Ly6g6e*, which is highly expressed in Chrna5+ neurons, is also a marker of subplate neurons.^69^ Our study is the first to identify enhanced cholinergic activation regulated by *Chrna5* and lynx-gene expression in subplate/L6b neurons. Subplate neurons have recently been found to strongly regulate cortical output through their intracortical connections.^70^^,^^71^ Enhanced cholinergic activation in these neurons will have different consequences for prefrontal processing, challenging the popular conception that cholinergic modulation of attention occurs only through top-down control of thalamic input by L6CT neurons.

### Molecular determinants of nicotinic receptor properties in Chrna5+ neurons

Our transcriptomic analysis revealed enhanced expression of GPI-anchored lynx prototoxin genes *Ly6g6e*, *Lypd1*, and *Lypd6b* in Chrna5+ neurons (Figure 5). Lynx proteins are well-known modulators of nicotinic receptor properties and trafficking,^38^^,^^40^ but most of the insight into their actions comes from heterologous cell systems, deletion, and overexpression experiments. Their effects on nicotinic receptors in their native environment are unclear. In expression systems, Ly6g6e potentiates α4β2 nicotinic responses, slowing their desensitization;^36^ predicting cholinergic responses in Chrna5+ neurons would be resistant to desensitization as has been implied by *Chrna5*-deletion work.^16^ In contrast, Lynx2 (*Lypd1*) is a predicted negative modulator that can increase desensitization of α4β2 nicotinic receptors.^72^ Lynx2 acts intracellularly to reduce surface expression of α4β2 nicotinic receptors,^36^ but may preferentially act on lower-affinity (α4)_3_(β2)_2_ receptors^61^^,^^73^ and indirectly promote expression of high-affinity (α4)_2_(β2)_2_α5 nicotinic receptors. The effect of Lypd6b on (α4)_2_(β2)_2_α5 nicotinic receptors is yet to be determined and may further contribute to the complex control of Chrna5+ nicotinic responses.^74^ In addition, Lynx1, a well-known negative modulator of α4β2 nicotinic receptors,^39^^,^^75^^,^^76^^,^^77^ is also expressed in Chrna5+ neurons. Consistent with such complex lynx regulation, our experiments confirmed that removing GPI-anchored lynx proteins increases nicotinic response onset and amplitude potentially due to removal of Lynx1. In contrast, exogenous application of recombinant Ly6g6e had different effects in Chrna5+ and Syt6+ neurons, consistent with cell type-specific lynx modulation in Chrna5+ neurons predicted by transcriptomics.

### Functional consequences

The effects of lynx proteins on nicotinic receptor function have so far been determined by heterologous expression systems,^36^ knockout studies,^72^^,^^78^ exogenous application of recombinant water-soluble lynx proteins,^79^^,^^80^ and viral manipulation of expression in the brain.^39^^,^^81^ We advance this field by revealing, in native tissue, complex endogenous regulation of optogenetic nicotinic responses by multiple GPI-anchored lynx proteins. Inhibitory lynx expression and high levels of acetylcholinesterase in Chrna5+ neurons suggest that their responses are restrained and our experiments likely underestimated their nicotinic receptor function. These responses could be dramatically enhanced when acetylcholinesterase and inhibitory lynx modulation is reduced through other signaling mechanisms. Such flexible tuning of nicotinic responses by lynx prototoxins in Chrna5+ neurons can provide greater dynamic range and poises them to be key players during attentional processing (Figure 5). A recent study found that preventing developmental increase in Lynx1 expression in corticocortical neurons by viral knockdown led to altered cortical connectivity and impaired attention.^39^ Thus cell type-specific changes in lynx expression during development are critical for maturation of attention circuits. It is of interest to examine such changes during development in Chrna5+ neurons and how they differ from Syt6+ neurons.

### Caveats and limitations of the study

Subplate-marker genes and localization of subplate cells can vary across cortical regions, and they have been better studied in sensory cortical regions.^82^^,^^83^^,^^84^ Our optophysiological, anatomical, and transcriptomic data show Chrna5+ acetylcholine super-responder neurons to be found in layer 6b both in the primary somatosensory cortex and PFC (Figures S2 and S3). However, an outstanding question is whether transcriptomic cell classes such as L5/6NP and L6b, which express multiple subplate-marker genes, correlate with early developmental subplate origin. Anatomical evidence alone is insufficient for this purpose. For example, while the distinct transcriptomic subclasses L6b and L5/6NP show overlapping spatial distribution in deep layers of the motor cortex,^85^^,^^86^ these subclasses possess distinct epigenetic signatures^87^ whose implications are yet to be understood. It will be necessary for future work to examine whether transcriptomic/epigenetically defined cell classes (L5/6NP, L6b) expressing subplate markers also show distinct early developmental origin. Lack of labeling strategies has limited efforts to characterize these neuronal subclasses and verify their developmental subplate identity. *Chrna5*-Cre mice will be an important tool to rectify this as they consistently label these conserved subclasses across multiple cortical regions. Another caveat of our study is the perturbation of lynx prototoxins using chemical strategies. While we have demonstrated cell type-specific effects of recombinant ly6g6e, it remains to be seen whether cell type-specific expression of ly6g6e or other lynx prototoxins is necessary for nicotinic modulation. Future work is needed to genetically manipulate lynx prototoxins in specified cell populations.

### Summary of advances

Our study reveals a distinct group of “acetylcholine super-responder” neurons in the prefrontal cortex identified by *Chrna5*-expression that constitute subplate neurons vital for cortical development. We identify that their high-affinity α5 subunit-containing nicotinic receptors are under complex regulation by several lynx prototoxins and acetylcholinesterase. *Chrna5*-Cre mice are a valuable tool for future studies examining the *in vivo* role of these specialized neurons.

## STAR★Methods

### Key resources table

**Table:** 

REAGENT or RESOURCE	SOURCE	IDENTIFIER
**Chemicals, peptides, and recombinant proteins**		

Acetylcholine	Sigma-Aldrich	A9101
Atropine	Sigma-Aldrich	A0132
Dihydro-β-erythroidine hydrobromide	Tocris	2349
Mecamylamine	Tocris	2843
m-3M3FBS	Tocris	1941
o-3M3FBS	Tocris	1942
2-Hydroxypropyl-*β*-cyclodextrin	Tocris	0708
recombinant Ly6g6e	Creative Biomart	N/A

**Deposited data**		

ACA and MOP- SMART-SEQ (2018)	Tasic et al., 2018^49^	Allen scRNAseq database
Whole cortex & Hippocampus - SMART-SEQ 2019 dataset updated with 10X-SMART-SEQ taxonomy (2021)	Yao et al., 2021^47^	Allen scRNAseq database

**Experimental models: organisms/strains**		

*Syt6*-Cre	GENSAT Project at Rockefeller University	RRID:MMRRC 037416-UCD
*Chrna5*-Cre	Gift from Dr. Eric E Turner	RRID:MGI:6201564
Ai96	The Jackson Laboratory	RRID:IMSR_JAX:024106
*Syt6*-EGFP EL71	GENSAT Project at Rockefeller University	RRID:MMRRC 010557-UCD
ChAT-ChR2	The Jackson Laboratory	RRID:IMSR_JAX:014546

**Software and algorithms**		

Seurat package	Hao et al., 2021^88^ Stuart et al., 2019^89^ Butler et al., 2018^90^	Seurat V4

### Resource availability

#### Lead contact

Further information and requests for resources and reagents should be directed to Dr. Evelyn Lambe (evelyn.lambe@utoronto.ca).

#### Materials availability

This study did not generate new unique reagents.

### Experimental model and subject details

*Syt6*-Cre^/+^GCaMP6s^/+^ and *Chrna5*-Cre^/+^GCaMP6s^/+^ mice used for Ca^2+^ imaging were obtained by crossing *Chrna5*-Cre (Gift from Dr. Eric Turner) and *Syt6*-Cre mice (*Syt6*-Cre KI148, RRID:MMRRC 037416-UCD,^91^) respectively, with Ai96 mice (JAX: 024106). For electrophysiological recordings of labeled Chrna5+ and Syt6+ neurons, we used *Chrna5*-Cre^/+^Ai14^/+^, and *Syt6*-Cre^/+^Ai14^/+^ mice respectively. *Syt6*-EGFP^/+^ mice were additionally used for few experiments (*Syt6*-EGFP EL71, RRID:MMRRC 010557-UCD,^92^).

Triple transgenic mice labeling both *Chrna5* and *Syt6-*expressing neurons with EGFP in Syt6+ neurons and tdTomato in Chrna5+ neurons were used to examine the overlap between the two cell types. *Syt6*-EGFP and Ai14 mice^93^ were bred together and the offspring were crossed with *Chrna5*-Cre mice to generate *Chrna5*-Cre^/+^Ai14^/+^*Syt6*-EGFP^/+^ mice used for these experiments. A set of experiments measuring optogenetic cholinergic responses was also performed in ChAT-ChR2 (ChAT^/+^) mice (JAX: 014546). To examine optogenetic cholinergic responses in labeled Chrna5 and Syt6 cell populations, the respective Cre lines were crossed with ChAT^/+^Ai14^/+^ mice to generate *Chrna5*-Cre^/+^Ai14^/+^ChAT^/+^ and *Syt6*-Cre^/+^Ai14^/+^ChAT^/+^ mice.

All animals were bred on a C57BL/6 background, except *Syt6*-EGFP which were Black Swiss. Adult male and female animals age >P60 were used in the study. Mice were separated based on sex after weaning at P21 and group-housed (2–4 mice per cage). Animals had *ad libitum* access to food and water and were on a 12-h light/dark cycle with lights on at 7 AM. Guidelines of the Canadian Council on Animal Care were followed, and all experimental procedures were approved by the Faculty of Medicine Animal Care Committee at the University of Toronto. 42 mice were used for the entire study, with similar numbers of males and females.

### Method details

#### Brain slicing and electrophysiology

Slicing and electrophysiology followed procedures described previously.^16^ An intraperitoneal injection of chloral hydrate (400 mg/kg) was given to anesthetize mice prior to decapitation. The brain was rapidly extracted in ice cold sucrose ACSF (254 mM sucrose, 10 mM D-glucose, 26 mM NaHCO_3_, 2 mM CaCl_2_, 2 mM MgSO_4_, 3 mM KCl and 1.25 mM NaH_2_PO_4_). 400 μm thick coronal slices of prefrontal cortex (Bregma 2.2 - 1.1) were obtained on a Dosaka linear slicer (SciMedia, Costa Mesa, CA, USA). Slices were left to recover for at least 2 hours in oxygenated (95% O_2_, 5% CO_2_) ACSF (128 mM NaCl, 10 mM D-glucose, 26 Mm NaHCO_3_, 2 mM CaCl_2_, 2 mM MgSO_4_, 3 Mm KCl, and 1.25 mM NaH_2_PO_4_) at 30°C before being used for electrophysiology or two-photon Ca^2+^ imaging. Brain slices were transferred to the stage of a BX51WI microscope (Olympus, Tokyo, Japan) and perfused with oxygenated ACSF at 30°C. Recording electrodes (2–4 MΩ) containing patch solution (120 mM potassium gluconate, 5 mM KCl, 10 mM HEPES, 2 mM MgCl_2_, 4 mM K_2_-ATP, 0.4 mM Na_2_-GTP and 10 mM sodium phosphocreatine, pH adjusted to 7.3 using KOH) were used to patch pyramidal neurons in layer 6 - 6b based on morphology and proximity to white matter. Only regular spiking neurons were included. Multiclamp 700B amplifier at 20 kHz with Digidata 1440A and pClamp 10.7 software (Molecular devices) were used for data acquisition. All recordings were compensated for liquid junction potential (14 mV). Voltage-clamp responses were examined at −75 mV and in current-clamp at rest or starting from −70 mV.

#### Optogenetics

5 ms pulses of blue light (473 nm) were delivered through the 60X objective lens with an LED (Thorlabs, 2 mW) to excite channelrhodopsin containing cholinergic fibers. Pattern of stimulation was as in a previous study, with 8 pulses of light delivered in a frequency accommodating manner.^16^

#### Pharmacology

Acetylcholine (1mM, Sigma) was used to exogenously stimulate cholinergic receptors. Atropine (200 nM, Sigma) and Dihydro-β-erythroidine (DHBE, 10 μM, Tocris) were used to competitively block muscarinic receptors and β2 subunit-containing nicotinic receptors respectively. Mecamylamine (5 μM, Tocris) was used to further non-competitively block nicotinic receptors. Phospholipase C activator m-3M3FBS (25 μM, Tocris) was used to cleave GPI-anchored Lynx prototoxins and the inactive ortholog o-3M3FBS (25 μM, Tocris) was used as a control.^55^ Cyclodextrin (1 mM, Tocris) was included in a small subset of experiments to improve solubility of 3M3FBS compounds, but no further improvement in efficacy was observed. Water soluble recombinant Ly6g6e (0.5 mg/ml) was obtained by custom purification (Creative Biomart) and used for exogenous application at 1: 1000 and 3:1000 dilution. Effects on nicotinic receptors were not distinguishable between the two different protein concentrations. Only freshly thawed protein aliquots were used for experiments.

#### Two-photon imaging

Two-photon imaging of GCaMP6s Ca^2+^ signals in L6 neurons was performed using a 60× water-immersion objective with 0.90 numerical aperture using an Olympus Fluoview FV1000 microscope and a Titanium-Sapphire laser sapphire laser (Newport) at 930nm. Images were sampled at 512 x 512 pixels (2.4 pixels/μm) at a frame rate of 0.9 Hz. Following a 2-minute washout period for this initial application, GCaMP6s Ca^2+^ signals were measured in response to acetylcholine (1 mM, 15 s). The cellular responses to acetylcholine were measured at baseline, then after application of competitive nicotinic receptor antagonist DHBE (10 μM, 10 min), and again after the addition of muscarinic antagonist atropine (200 nM, 10 min).

Dual color two-photon imaging (910 nm excitation, using 570 nm dichroic mirror with green (540–595 nm) and red (570–620 nm) filters) was performed in brain slices of triple transgenic *Chrna5*-Cre^/+^Ai14^/+^*Syt6*-EGFP^/+^ mice to examine overlap in fluorescent reporter expression between Chrna5+ and Syt6+ neurons. Z-stacks of 30 frames acquired in 1-μm steps were taken in layer 6 of mPFC slices and the maximum projection used to count cells with the cell counter feature in Fiji. A set of mPFC brain slices from *Chrna5*-Cre^/+^Ai14^/+^*Syt6*-EGFP^/+^ mice were also fixed and mounted for confocal imaging with LSM880 (Leica) microscope.

#### Single cell RNAseq analysis

Single cell RNAseq data for Anterior Cingulate Cortex (ACA) of adult mice was taken from the ACA and MOP Smart-Seq (2018) database, with cell-type annotations from Whole cortex & Hippocampus Smart-Seq (2019) database from the Allen Institute for Brain Science at https://portal.brain-map.org/atlases-and-data/rnaseq.^47^^,^^49^ Single cell analysis was performed using the R package Seurat (v 4.04).^88^^,^^89^^,^^90^ Layer 5 and 6 glutamatergic neurons were selected and sorted into three cell classes based on their expression of *Chrna5* and *Syt6* genes: those expressing only *Chrna5* (Chrna5+, n = 243), only *Syt6* (Syt6+, n = 834), or both *Chrna5* and *Syt6* (Chrna5+Syt6+, n = 564). Expression (copies per million) greater than zero was used as the threshold. 781 cells did not express either *Chrna5* or *Syt6* and were not used in subsequent analyses*.* Rare cell types with fewer than 10 cells per group are not shown in the heatmap in Figure 5 but are included for the differential expression analysis. The FindMarkers function in Seurat was used to identify genes differentially expressed between the Chrna5+ and Chrna5+Syt6+ populations. Adjusted p value < 0.05 was used as the cutoff for identifying differentialy expressed genes. Similar approaches were used to perform the same analyses in multiple cortical regions (Figure S5). L5-6 glutamatergic cells cells in ACA, MOp, SSp, and VISp were taken from the Whole cortex & Hippocampus Smart-Seq (2019) dataset.

### Quantification and statistical analysis

Analysis of electrophysiological data was conducted on Clampfit 10.7 and Axograph. Rising slope was measured by fitting a line to the first 50ms of the optogenetic cholinergic responses. Cholinergic response magnitude in voltage clamp was determined by peak current (picoamperes) and charge transfer (picocoulombs) measured by the area under the current response for 1 second.

GCaMP6s imaging data were extracted using the multi-measure feature in Fiji. Maximum projections across time for each experiment were first used to identify acetylcholine-responsive cells and add them to the ROI manager, then fluorescence intensity at all timepoints for each cell was measured. Fluorescence was normalized to the background fluorescence averaged over the first 10 frames. Area under the peak (AUP) of the signal after baseline correction was used to quantify the magnitude of cells’ response to acetylcholine. Percentage response remaining after DHBE and DHBE+Atropine was calculated from the cell’s AUP before and after the blockers. The statistical analysis for electrophysiological recordings and calcium imaging treated each cell as independent with several cells recorded from each slice.

GraphPad Prism 8 was used for statistical analysis and plotting graphs. Bar graphs depicting mean with standard error and boxplots with median and quartiles are shown. Effect sizes are reported as Cohen’s d for major results.^94^ Unpaired t-tests or Mann-Whitney tests were used when comparing response properties between cell types, and paired t-test or Wilcoxon test to quantify effect of pharmacological manipulations within cells. Kolmogorov-Smirnov and Fisher’s exact tests were used to compare cumulative distributions and proportions of cells respectively.

## Data Availability

This paper analyzes existing, publicly available single cell RNAseq data. The links for the Allen Institute scRNAseq database are provided in the key resources table. The paper does not report original code. Any additional information required to reanalyze the data reported in this paper is available from the lead contact upon request.

## Supplemental information

Document S1. Figures S1–S5 and Tables S1–S3

Table S4. Excel file containing all differentially expressed genes between Chrna5+ and Chrna5+Syt6+ neurons with adjusted p value <0.05, related to Table 1

## References

[1] Dalley J.W., Cardinal R.N., Robbins T.W. (2004). Prefrontal executive and cognitive functions in rodents: neural and neurochemical substrates. Neurosci. Biobehav. Rev..

[2] McGaughy J., Dalley J.W., Morrison C.H., Everitt B.J., Robbins T.W., Robbins T.W. (2002). Selective behavioral and neurochemical effects of cholinergic lesions produced by intrabasalis infusions of 192 IgG-saporin on attentional performance in a five-choice serial reaction time task. J. Neurosci..

[3] Dalley J.W., Theobald D.E., Bouger P., Chudasama Y., Cardinal R.N., Robbins T.W. (2004). Cortical cholinergic function and deficits in visual attentional performance in rats following 192 IgG-saporin-induced lesions of the medial prefrontal cortex. Cerebr. Cortex.

[4] Gritton H.J., Howe W.M., Mallory C.S., Hetrick V.L., Berke J.D., Sarter M. (2016). Cortical cholinergic signaling controls the detection of cues. Proc. Natl. Acad. Sci. USA.

[5] Briggs F., Usrey W.M. (2008). Emerging views of corticothalamic function. Curr. Opin. Neurobiol..

[6] Voigts J., Deister C.A., Moore C.I. (2020). Layer 6 ensembles can selectively regulate the behavioral impact and layer-specific representation of sensory deviants. Elife.

[7] Spellman T., Svei M., Kaminsky J., Manzano-Nieves G., Liston C. (2021). Prefrontal deep projection neurons enable cognitive flexibility via persistent feedback monitoring. Cell.

[8] Kassam S.M., Herman P.M., Goodfellow N.M., Alves N.C., Lambe E.K. (2008). Developmental excitation of corticothalamic neurons by nicotinic acetylcholine receptors. J. Neurosci..

[9] Sparks D.W., Tian M.K., Sargin D., Venkatesan S., Intson K., Lambe E.K. (2017). Opposing cholinergic and serotonergic modulation of layer 6 in prefrontal cortex. Front. Neural Circ..

[10] Venkatesan S., Jeoung H.-S., Chen T., Power S.K., Liu Y., Lambe E.K. (2020). Behavioral Pharmacology of the Cholinergic System.

[11] Winzer-Serhan U.H., Leslie F.M. (2005). Expression of α5 nicotinic acetylcholine receptor subunit mRNA during hippocampal and cortical development. J. Comp. Neurol..

[12] Wada E., McKinnon D., Heinemann S., Patrick J., Swanson L.W. (1990). The distribution of mRNA encoded by a new member of the neuronal nicotinic acetylcholine receptor gene family (α5) in the rat central nervous system. Brain Res..

[13] Bailey C.D.C., De Biasi M., Fletcher P.J., Lambe E.K. (2010). The nicotinic acetylcholine receptor alpha5 subunit plays a key role in attention circuitry and accuracy. J. Neurosci..

[14] Tian M.K., Bailey C.D.C., De Biasi M., Picciotto M.R., Lambe E.K. (2011). Plasticity of prefrontal attention circuitry: upregulated muscarinic excitability in response to decreased nicotinic signaling following deletion of α5 or β2 subunits. J. Neurosci..

[15] Howe W.M., Brooks J.L., Tierney P.L., Pang J., Rossi A., Young D., Dlugolenski K., Guillmette E., Roy M., Hales K., Kozak R. (2018). α5 nAChR modulation of the prefrontal cortex makes attention resilient. Brain Struct. Funct..

[16] Venkatesan S., Lambe E.K. (2020). Chrna5 is essential for a rapid and protected response to optogenetic release of endogenous acetylcholine in prefrontal cortex. J. Neurosci..

[17] Han W., Zhang T., Ni T., Zhu L., Liu D., Chen G., Lin H., Chen T., Guan F. (2019). Relationship of common variants in CHRNA5 with early-onset schizophrenia and executive function. Schizophr. Res..

[18] Bierut L.J., Stitzel J.A., Wang J.C., Hinrichs A.L., Grucza R.A., Xuei X., Saccone N.L., Saccone S.F., Bertelsen S., Fox L. (2008). Variants in the nicotinic receptors alter the risk for nicotine dependence. Am. J. Psychiatr..

[19] Schuch J.B., Polina E.R., Rovaris D.L., Kappel D.B., Mota N.R., Cupertino R.B., Silva K.L., Guimarães-da-Silva P.O., Karam R.G., Salgado C.A.I. (2016). Pleiotropic effects of Chr15q25 nicotinic gene cluster and the relationship between smoking, cognition and ADHD. J. Psychiatr. Res..

[20] Maskos U. (2020). The nicotinic receptor alpha5 coding polymorphism rs16969968 as a major target in disease: functional dissection and remaining challenges. J. Neurochem..

[21] Scholze P., Huck S. (2020). The α5 nicotinic acetylcholine receptor subunit differentially modulates α4β2∗ and α3β4∗ receptors. Front. Synaptic Neurosci..

[22] Prevost M.S., Bouchenaki H., Barilone N., Gielen M., Corringer P.J. (2020). Concatemers to re-investigate the role of α5 in α4β2 nicotinic receptors. Cell. Mol. Life Sci..

[23] Thomson A.M. (2010). Neocortical layer 6, a review. Front. Neuroanat..

[24] Briggs F. (2010). Organizing principles of cortical layer 6. Front. Neural Circ..

[25] Sorensen S.A., Bernard A., Menon V., Royall J.J., Glattfelder K.J., Desta T., Hirokawa K., Mortrud M., Miller J.A., Zeng H. (2015). Correlated gene expression and target specificity demonstrate excitatory projection neuron diversity. Cerebr. Cortex.

[26] Heath C.J., King S.L., Gotti C., Marks M.J., Picciotto M.R. (2010). Cortico-thalamic connectivity is vulnerable to nicotine exposure during early postnatal development through α4/β2/α5 nicotinic acetylcholine receptors. Neuropsychopharmacology.

[27] Nakayama H., Ibañez-Tallon I., Heintz N. (2018). Cell-type-specific contributions of medial prefrontal neurons to flexible behaviors. J. Neurosci..

[28] Vaasjo L.O., Han X., Thurmon A.N., Tiemroth A.S., Berndt H., Korn M., Figueroa A., Reyes R., Feliciano-Ramos P.A., Galazo M.J. (2022). Characterization and manipulation of Corticothalamic neurons in associative cortices using Syt6-Cre transgenic mice. J. Comp. Neurol..

[29] Tian M.K., Bailey C.D.C., Lambe E.K. (2014). Cholinergic excitation in mouse primary vs. associative cortex: region-specific magnitude and receptor balance. Eur. J. Neurosci..

[30] Ables J.L., Görlich A., Antolin-Fontes B., Wang C., Lipford S.M., Riad M.H., Ren J., Hu F., Luo M., Kenny P.J. (2017). Retrograde inhibition by a specific subset of interpeduncular α5 nicotinic neurons regulates nicotine preference. Proc. Natl. Acad. Sci. USA.

[31] Morton G., Nasirova N., Sparks D.W., Brodsky M., Sivakumaran S., Lambe E.K., Turner E.E. (2018). Chrna5-expressing neurons in the interpeduncular nucleus mediate aversion primed by prior stimulation or nicotine exposure. J. Neurosci..

[32] Hoerder-Suabedissen A., Molnár Z. (2013). Molecular diversity of early-born subplate neurons. Cerebr. Cortex.

[33] Luhmann H.J., Kirischuk S., Kilb W. (2018). The superior function of the subplate in early neocortical development. Front. Neuroanat..

[34] Kanold P.O., Luhmann H.J. (2010). The subplate and early cortical circuits. Annu. Rev. Neurosci..

[35] Miwa J.M., Anderson K.R., Hoffman K.M. (2019). Lynx prototoxins: roles of endogenous mammalian neurotoxin-like proteins in modulating nicotinic acetylcholine receptor function to influence complex biological processes. Front. Pharmacol..

[36] Wu M., Puddifoot C.A., Taylor P., Joiner W.J. (2015). Mechanisms of inhibition and potentiation of α4β2 nicotinic acetylcholine receptors by members of the Ly6 protein family. J. Biol. Chem..

[37] Demars M.P., Morishita H. (2014). Cortical parvalbumin and somatostatin GABA neurons express distinct endogenous modulators of nicotinic acetylcholine receptors. Mol. Brain.

[38] Miwa J.M. (2021). Lynx1 prototoxins: critical accessory proteins of neuronal nicotinic acetylcholine receptors. Curr. Opin. Pharmacol..

[39] Falk E.N., Norman K.J., Garkun Y., Demars M.P., Im S., Taccheri G., Short J., Caro K., Mccraney S.E., Cho C. (2021). Nicotinic regulation of local and long-range input balance drives top-down attentional circuit maturation. Sci. Adv..

[40] Anderson K.R., Hoffman K.M., Miwa J.M. (2020). Modulation of cholinergic activity through lynx prototoxins: implications for cognition and anxiety regulation. Neuropharmacology.

[41] Paxinos G., Franklin K. (2001).

[42] Nectow A.R., Moya M.V., Ekstrand M.I., Mousa A., McGuire K.L., Sferrazza C.E., Field B.C., Rabinowitz G.S., Sawicka K., Liang Y. (2017). Rapid molecular profiling of defined cell types using viral TRAP. Cell Rep..

[43] Harris J.A., Mihalas S., Hirokawa K.E., Whitesell J.D., Choi H., Bernard A., Bohn P., Caldejon S., Casal L., Cho A. (2019). Hierarchical organization of cortical and thalamic connectivity. Nature.

[44] Heintz N. (2004). Gene expression nervous system atlas (GENSAT). Nat. Neurosci..

[45] Webster J.C., Francis M.M., Porter J.K., Robinson G., Stokes C., Horenstein B., Papke R.L. (1999). Antagonist activities of mecamylamine and nicotine show reciprocal dependence on beta subunit sequence in the second transmembrane domain. Br. J. Pharmacol..

[46] Papke R.L., Stokes C., Muldoon P., Imad Damaj M. (2013). Similar activity of mecamylamine stereoisomers in vitro and in vivo. Eur. J. Pharmacol..

[47] Yao Z., van Velthoven C.T.J., Nguyen T.N., Goldy J., Sedeno-Cortes A.E., Baftizadeh F., Bertagnolli D., Casper T., Chiang M., Crichton K. (2021). A taxonomy of transcriptomic cell types across the isocortex and hippocampal formation. Cell.

[48] Tasic B., Menon V., Nguyen T.N., Kim T.K., Jarsky T., Yao Z., Levi B., Gray L.T., Sorensen S.A., Dolbeare T. (2016). Adult mouse cortical cell taxonomy revealed by single cell transcriptomics. Nat. Neurosci..

[49] Tasic B., Yao Z., Graybuck L.T., Smith K.A., Nguyen T.N., Bertagnolli D., Goldy J., Garren E., Economo M.N., Viswanathan S. (2018). Shared and distinct transcriptomic cell types across neocortical areas. Nature.

[50] Yang D., Günter R., Qi G., Radnikow G., Feldmeyer D. (2020). Muscarinic and nicotinic modulation of neocortical layer 6A synaptic microcircuits is cooperative and cell-specific. Cerebr. Cortex.

[51] Ghezzi F., Marques-Smith A., Anastasiades P.G., Lyngholm D., Vagnoni C., Rowett A., Parameswaran G., Hoerder-Suabedissen A., Nakagawa Y., Molnar Z., Butt S.J. (2021). Non-canonical role for lpar1-egfp subplate neurons in early postnatal mouse somatosensory cortex. Elife.

[52] Wess J.M., Isaiah A., Watkins P.V., Kanold P.O. (2017). Subplate neurons are the first cortical neurons to respond to sensory stimuli. Proc. Natl. Acad. Sci. USA.

[53] Hansen J.Y., Markello R.D., Tuominen L., Nørgaard M., Kuzmin E., Palomero-Gallagher N., Dagher A., Misic B. (2022). Correspondence between gene expression and neurotransmitter receptor and transporter density in the human brain. Neuroimage.

[54] Abbondanza A., Ribeiro Bas I., Modrak M., Capek M., Minich J., Tyshkevich A., Naser S., Rangotis R., Houdek P., Sumova A. (2022). Nicotinic acetylcholine receptors expressed by striatal interneurons inhibit striatal activity and control striatal-dependent behaviors. J. Neurosci..

[55] Bae Y.S., Lee T.G., Park J.C., Hur J.H., Kim Y., Heo K., Kwak J.Y., Suh P.G., Ryu S.H. (2003). Identification of a compound that directly stimulates phospholipase C activity. Mol. Pharmacol..

[56] Krjukova J., Holmqvist T., Danis A.S., Åkerman K.E.O., Kukkonen J.P. (2004). Phospholipase C activator m-3M3FBS affects Ca2+ homeostasis independently of phospholipase C activation. Br. J. Pharmacol..

[57] Sturgeon R.M., Magoski N.S. (2018). A closely associated phospholipase C regulates cation channel function through phosphoinositide hydrolysis. J. Neurosci..

[58] Horowitz L.F., Hirdes W., Suh B.-C., Hilgemann D.W., Mackie K., Hille B. (2005). Phospholipase C in living cells activation, inhibition, Ca2+ requirement, and regulation of M current. J. Gen. Physiol..

[59] Kulbatskii D., Shenkarev Z., Bychkov M., Loktyushov E., Shulepko M., Koshelev S., Povarov I., Popov A., Peigneur S., Chugunov A. (2021). Human three-finger protein Lypd6 is a negative modulator of the cholinergic system in the brain. Front. Cell Dev. Biol..

[60] Arvaniti M., Jensen M.M., Soni N., Wang H., Klein A.B., Thiriet N., Pinborg L.H., Muldoon P.P., Wienecke J., Imad Damaj M. (2016). Functional interaction between Lypd6 and nicotinic acetylcholine receptors. J. Neurochem..

[61] Kuryatov A., Onksen J., Lindstrom J. (2008). Roles of accessory subunits in α4β2∗ nicotinic receptors. Mol. Pharmacol..

[62] Harris J.A., Hirokawa K.E., Sorensen S.A., Gu H., Mills M., Ng L.L., Bohn P., Mortrud M., Ouellette B., Kidney J. (2014). Anatomical characterization of Cre driver mice for neural circuit mapping and manipulation. Front. Neural Circ..

[63] Sundberg S.C., Lindström S.H., Sanchez G.M., Granseth B. (2018). Cre-expressing neurons in visual cortex of Ntsr1-Cre GN220 mice are corticothalamic and are depolarized by acetylcholine. J. Comp. Neurol..

[64] Molnár Z., Luhmann H.J., Kanold P.O. (2020). Transient cortical circuits match spontaneous and sensory-driven activity during development. Science.

[65] Marx M., Qi G., Hanganu-Opatz I.L., Kilb W., Luhmann H.J., Feldmeyer D. (2017). Neocortical layer 6B as a remnant of the subplate - a morphological comparison. Cerebr. Cortex.

[66] Mechawar N., Descarries L. (2001). The cholinergic innervation develops early and rapidly in the rat cerebral cortex: a quantitative immunocytochemical study. Neuroscience.

[67] Hoerder-Suabedissen A., Wang W.Z., Lee S., Davies K.E., Goffinet A.M., Rakić S., Parnavelas J., Reim K., Nicolić M., Paulsen O., Molnár Z. (2009). Novel markers reveal subpopulations of subplate neurons in the murine cerebral cortex. Cerebr. Cortex.

[68] Tiong S.Y.X., Oka Y., Sasaki T., Taniguchi M., Doi M., Akiyama H., Sato M. (2019). Kcnab1 is expressed in subplate neurons with unilateral long-range inter-areal projections. Front. Neuroanat..

[69] Hoerder-Suabedissen A., Oeschger F.M., Krishnan M.L., Belgard T.G., Wang W.Z., Lee S., Webber C., Petretto E., Edwards A.D., Molnár Z. (2013). Expression profiling of mouse subplate reveals a dynamic gene network and disease association with autism and schizophrenia. Proc. Natl. Acad. Sci. USA.

[70] Zolnik T.A., Ledderose J., Toumazou M., Trimbuch T., Oram T., Rosenmund C., Eickholt B.J., Sachdev R.N.S., Larkum M.E. (2020). Layer 6b is driven by intracortical long-range projection neurons. Cell Rep..

[71] Egger R., Narayanan R.T., Guest J.M., Bast A., Udvary D., Messore L.F., Das S., de Kock C.P.J., Oberlaender M. (2020). Cortical output is gated by horizontally projecting neurons in the deep layers. Neuron.

[72] Tekinay A.B., Nong Y., Miwa J.M., Lieberam I., Ibanez-Tallon I., Greengard P., Heintz N. (2009). A role for LYNX2 in anxiety-related behavior. Proc. Natl. Acad. Sci. USA.

[73] Nichols W.A., Henderson B.J., Yu C., Parker R.L., Richards C.I., Lester H.A., Miwa J.M. (2014). Lynx1 shifts α4β2 nicotinic receptor subunit stoichiometry by affecting assembly in the endoplasmic reticulum. J. Biol. Chem..

[74] Ochoa V., George A.A., Nishi R., Whiteaker P. (2016). The prototoxin LYPD6B modulates heteromeric a3β4-containing nicotinic acetylcholine receptors, but not α7 homomers. Faseb. J..

[75] Miwa J.M., Ibaňez-Tallon I., Crabtree G.W., Sánchez R., Šali A., Role L.W., Heintz N. (1999). lynx1, an endogenous toxin-like modulator of nicotinic acetylcholine receptors in the mammalian CNS. Neuron.

[76] Ibañez-Tallon I., Miwa J.M., Wang H.L., Adams N.C., Crabtree G.W., Sine S.M., Heintz N. (2002). Novel modulation of neuronal nicotinic acetylcholine receptors by association with the endogenous prototoxin lynx1. Neuron.

[77] Morishita H., Miwa J.M., Heintz N., Hensch T.K. (2010). Lynx1, a cholinergic brake, limits plasticity in adult visual cortex. Science.

[78] Sherafat Y., Chen E., Lallai V., Bautista M., Fowler J.P., Chen Y.C., Miwa J., Fowler C.D. (2021). Differential expression patterns of Lynx proteins and involvement of Lynx1 in prepulse inhibition. Front. Behav. Neurosci..

[79] Shenkarev Z.O., Shulepko M.A., Bychkov M.L., Kulbatskii D.S., Shlepova O.v., Vasilyeva N.A., Andreev-Andrievskiy A.A., Popova A.S., Lagereva E.A., Loktyushov E.v. (2020). Water-soluble variant of human Lynx1 positively modulates synaptic plasticity and ameliorates cognitive impairment associated with α7-nAChR dysfunction. J. Neurochem..

[80] Thomsen M.S., Arvaniti M., Jensen M.M., Shulepko M.A., Dolgikh D.A., Pinborg L.H., Härtig W., Lyukmanova E.N., Mikkelsen J.D. (2016). Lynx1 and Aβ1–42 bind competitively to multiple nicotinic acetylcholine receptor subtypes. Neurobiol. Aging.

[81] Sadahiro M., Demars M.P., Burman P., Yevoo P., Zimmer A., Morishita H. (2020). Activation of somatostatin interneurons by nicotinic modulator Lypd6 enhances plasticity and functional recovery in the adult mouse visual cortex. J. Neurosci..

[82] Viswanathan S., Bandyopadhyay S., Kao J.P.Y., Kanold P.O. (2012). Changing microcircuits in the subplate of the developing cortex. J. Neurosci..

[83] Viswanathan S., Sheikh A., Looger L.L., Kanold P.O. (2017). Molecularly defined subplate neurons project both to thalamocortical recipient layers and thalamus. Cerebr. Cortex.

[84] Hoerder-Suabedissen A., Hayashi S., Upton L., Nolan Z., Casas-Torremocha D., Grant E., Viswanathan S., Kanold P.O., Clasca F., Kim Y., Molnár Z. (2018). Subset of cortical layer 6b neurons selectively innervates higher order thalamic nuclei in mice. Cerebr. Cortex.

[85] Zhang M., Eichhorn S.W., Zingg B., Yao Z., Cotter K., Zeng H., Dong H., Zhuang X. (2021). Spatially resolved cell atlas of the mouse primary motor cortex by MERFISH. Nature.

[86] Muñoz-Castañeda R., Zingg B., Matho K.S., Chen X., Wang Q., Foster N.N., Li A., Narasimhan A., Hirokawa K.E., Huo B. (2021). Cellular anatomy of the mouse primary motor cortex. Nature.

[87] Yao Z., Liu H., Xie F., Fischer S., Adkins R.S., Aldridge A.I., Ament S.A., Bartlett A., Behrens M.M., van den Berge K. (2021). A transcriptomic and epigenomic cell atlas of the mouse primary motor cortex. Nature.

[88] Hao Y., Hao S., Andersen-Nissen E., Mauck W.M., Zheng S., Butler A., Lee M.J., Wilk A.J., Darby C., Zager M. (2021). Integrated analysis of multimodal single-cell data. Cell.

[89] Stuart T., Butler A., Hoffman P., Hafemeister C., Papalexi E., Mauck W.M., Hao Y., Stoeckius M., Smibert P., Satija R. (2019). Comprehensive integration of single-cell data. Cell.

[90] Butler A., Hoffman P., Smibert P., Papalexi E., Satija R. (2018). Integrating single-cell transcriptomic data across different conditions, technologies, and species. Nat. Biotechnol..

[91] Gong S., Doughty M., Harbaugh C.R., Cummins A., Hatten M.E., Heintz N., Gerfen C.R. (2007). Targeting Cre recombinase to specific neuron populations with bacterial artificial chromosome constructs. J. Neurosci..

[92] Gong S., Zheng C., Doughty M.L., Losos K., Didkovsky N., Schambra U.B., Nowak N.J., Joyner A., Leblanc G., Hatten M.E., Heintz N. (2003). A gene expression atlas of the central nervous system based on bacterial artificial chromosomes. Nature.

[93] Madisen L., Zwingman T.A., Sunkin S.M., Wook Oh S., Zariwala H.A., Gu H., Ng L.L., Palmiter R.D., Hawrylycz M.J., Jones A.R. (2010). A robust and high-throughput Cre reporting and characterization system for the whole mouse brain. Nat. Neurosci..

[94] Ho J., Tumkaya T., Aryal S., Choi H., Claridge-Chang A. (2019). Moving beyond P values: data analysis with estimation graphics. Nat. Methods.

